# Sustained Nrf2 Overexpression-Induced Metabolic Deregulation Can Be Attenuated by Modulating Insulin/Insulin-like Growth Factor Signaling

**DOI:** 10.3390/cells12222650

**Published:** 2023-11-18

**Authors:** Sentiljana Gumeni, Maria Lamprou, Zoi Evangelakou, Maria S. Manola, Ioannis P. Trougakos

**Affiliations:** Department of Cell Biology and Biophysics, Faculty of Biology, National and Kapodistrian University of Athens, Panepistimiopolis, 15784 Athens, Greece; sgumeni@biol.uoa.gr (S.G.); mlamprou97@gmail.com (M.L.); zoievag@biol.uoa.gr (Z.E.); mmanola@biol.uoa.gr (M.S.M.)

**Keywords:** cncC, ImpL2, insulin/insulin-like growth factors, metformin, Nrf2

## Abstract

The modulation of insulin/insulin-like growth factor signaling (IIS) is associated with altered nutritional and metabolic states. The *Drosophila* genome encodes eight insulin-like peptides, whose activity is regulated by a group of secreted factors, including Ecdysone-inducible gene L2 (*ImpL2*), which acts as a potent IIS inhibitor. We recently reported that cncC (cncC/Nrf2), the fly ortholog of Nrf2, is a positive transcriptional regulator of ImpL2, as part of a negative feedback loop aiming to suppress cncC/Nrf2 activity. This finding correlated with our observation that sustained cncC/Nrf2 overexpression/activation (*cncC^OE^*; a condition that signals organismal stress) deregulates IIS, causing hyperglycemia, the exhaustion of energy stores in flies’ tissues, and accelerated aging. Here, we extend these studies in *Drosophila* by assaying the functional implication of ImpL2 in *cncC^OE^*-mediated metabolic deregulation. We found that *ImpL2* knockdown (KD) in *cncC^OE^* flies partially reactivated IIS, attenuated hyperglycemia and restored tissue energetics. Moreover, *ImpL2* KD largely suppressed *cncC^OE^*-mediated premature aging. In support, pharmacological treatment of *cncC^OE^* flies with Metformin, a first-line medication for type 2 diabetes, restored (dose-dependently) IIS functionality and extended *cncC^OE^* flies’ longevity. These findings exemplify the effect of chronic stress in predisposition to diabetic phenotypes, indicating the potential prophylactic role of maintaining normal IIS functionality.

## 1. Introduction

Insulin/insulin-like growth factor (IGFs) signaling (IIS) is an evolutionary conserved pathway that modulates longevity across species [1], with insulin being the primary hormone in controlling the balance between anabolic and catabolic processes. The initial step of IIS involves the binding of insulin to its specific cell surface receptor (insulin receptor/InR) and activation of its tyrosine kinase activity, followed by the phosphorylation of a small subset of proteins, which are part of two major downstream pathways, namely the IRS-1/phosphatidylinositol 3-kinase (PI3K)/AKT and the SHC/RAS/mitogen-activated protein kinase [2,3]. InR and IGFs are highly conserved regulators of bioenergetic pathways and organismal growth in vertebrates and invertebrates [4]. Similarly, Insulin like peptides (Ilps) are evolutionary conserved across metazoans, sharing similar structural motifs, and their binding affinity in most invertebrates is limited to one single InR-like receptor [5]. The human genome encodes ten members of the insulin-like peptide family; the closely related insulin and IGF-I and -II and seven peptides related to relaxin [5]. The cellular actions of insulin include increased glucose (GLU) uptake into tissues, glycogen (GLY) synthesis, lipogenesis, and decreased gluconeogenesis (breakdown of certain noncarbohydrate precursors). Beyond its blood-GLU-lowering effect, insulin also regulates the expression of downstream transcriptional targets, such as transcription factors involved in the regulation of energy distribution and genes involved in IIS as part of a negative feedback regulation mechanism [6].

The *Drosophila* genome encodes eight Ilps (dIlp1-8) with pleiotropic functions, with dIlp2 showing the highest homology with mammalian insulin [7]. dIlps are expressed in different developmental stages mainly, but not exclusively, by the β-cell-like insulin producing cells (IPCs) of the brain, while their expression is tightly modulated by a group of secreted factors [8]. Among these factors, ImpL2 (Imaginal Morphogenesis Protein-Late 2), also known as ecdysone-inducible gene L2, is expressed in the fat body, corpora cardiaca, and IPCs, where it functions as a negative regulator of IIS by binding to dIlps and inhibiting their activity [9,10]. ImpL2 has been found to bind human IGF-1, IGF-2, and insulin with high affinity [11], and it was initially considered an ortholog of IGFBP [12,13]. However, recent studies by Roed and colleagues, analyzing the ImpL2 structure and its hormone-binding modes, have shown no apparent relation to any of the human IGFBPs [14]; thereby, this remains controversial.

*Nuclear factor erythroid 2-like 2*, or *Nrf2* (encoded by *NFE2L2* in humans), is a member of the stress-activated cap’n’collar (*cnc*) transcription factors’ family, and as a response to stressful stimuli, it regulates the expression of a broad range of genes that contain an enhancer sequence in their promoter regulatory regions, known as the antioxidant response element (ARE). In fact, Nrf2 is the master regulator of cellular antioxidant responses [15,16]. Under non-stressful conditions, Nrf2 activity is maintained at low levels by being constitutively targeted for proteasomal degradation by Kelch-like ECH-associated protein-1 (Keap1)-mediated ubiquitylation [17]. Recent data underline that, besides Keap1-mediated proteasomal degradation, many other cellular processes (including metabolic pathways) potentially control Nrf2 activity [18]. Therefore, Nrf2 has a diverse role in cellular dynamics, acting not only as a stress sensor but also as a regulator of lipids, carbohydrates, nucleotides, and amino acid metabolism; thus, Nrf2 basal activity levels should be tightly controlled to ensure cellular homeostasis.

We recently showed [19] that the sustained inducible overexpression (OE) of *cap’n’collar* isoform *C* (*cncC*; the ortholog of mammalian *Nrf2*) in adult *Drosophila* suppresses IIS, leading to metabolic deregulation and diabetes-like phenotypes; we also found that *ImpL2* gene expression is directly regulated by *cncC/Nrf2* [19]. Herein, we show that the genetic downregulation of *ImpL2* or pharmacological treatment with the anti-diabetic drug Metformin in *cncC^OE^* transgenic flies ameliorated *cncC^OE^* -mediated metabolic deregulation and increased flies’ health span.

## 2. Materials and Methods

### 2.1. Fly Stocks

Fly stocks were maintained at 25 °C, 60% relative humidity, and a 12 h light/dark cycle, and they fed a standard diet (6.4% rice flour, 5% tomato paste, 3.2% sugar, 0.8% yeast, 0.8% agar, 0.4% ethanol, and 0.4% propionic acid). The transgenic strains *w1118* (#5905) and RNAi *ImpL2* (#64936) were obtained from the Bloomington Stock Center. The UAS *cncC* (*cncC^OE^*) transgenic line [20] and the GeneSwitch Gal4 Tubulin (GSGal4Tub) flies were a gift from Profs D. Bohmann (University of Rochester, Rochester, NY, USA) and G. Sykiotis (Lausanne University, Switzerland). The conditional driver GSGal4Tub uses a modified GAL4 protein fused to a progesterone steroid receptor, allowing the regulation of its GAL4 activity by the synthetic progesterone analogue mifepristone (RU486) [21,22]. Thus, precise control of transgene expression timing and levels can be achieved by adding to flies’ culture medium different doses of RU486. GSGal4Tub was ubiquitously activated upon dietary administration of 320 μΜ RU486. Gonads display distinct aging rates and the regulation of proteostatic mechanisms compared to adult somatic tissues [23]; thus, in all presented experiments (unless otherwise indicated), only micro-dissected adult somatic tissues (head and thorax; equal numbers from mated male and female flies) were analyzed.

### 2.2. Flies’ Culture, Exposure to Compounds, and Longevity Assays

We employed the inducible driver GSGal4Tub to regulate the expression of target genes. Compounds were added in flies’ culture medium; doses and duration of flies’ exposure to compounds are indicated in figure and/or figure legends. RU486 (M8046) was purchased from Sigma Aldrich (St. Louis, MO, USA) and Metformin (317240) from Merck (Darmstadt, Germany).

The inducible ubiquitous (GSGal4Tub) transgene expression was used to overcome developmental lethality caused by cncC overexpression [19]. The GSGal4Tub driver was crossed with the following lines: RNAi *ImpL2*, *cncC^OE^*, RNAi *InR*, and/or the double-transgenic lines generated by crossing these lines. Flies were collected following hatching and maintained at 25 °C for all experiments.

For longevity studies, populations of 40 flies (equal number of males and females) were transferred to fresh medium every 3 days and deaths were scored daily. Survival curves were generated as the results of each viability experiment (in total, ~180–240 flies), from at least three different fly crosses. For statistical analysis, the Kaplan–Meier procedure and log-rank test (Mantel–Cox) were used; significance was accepted at *p* < 0.05.

### 2.3. Genomic DNA Extraction and Conventional PCR Analyses

To verify the establishment of transgenic flies carrying more than one transgene, genomic DNA from larvae or flies’ tissues was extracted with the Genomic DNA Kit (K0512, Thermo Fisher Scientific, Waltham, MA, USA). Conventional PCR analysis was then performed as described before [19]. Primers were designed using the primer-BLAST tool (http://www.ncbi.nlm.nih.gov/tools/primer-blast/, accessed on 12 February 2019) and were the following: Valium20-F:ACCAGCAACCAAGTAAATCAAC, Valium20-R:TAATCGTGTGTGATGCCTACC; cncC-F: TGGAATTGGGCACCCATGGCG, cncC-R: AGTTTGAGTACGTCGTTCAACA.

### 2.4. RNA Extraction, cDNA Synthesis, and Quantitative Real-Time PCR (Q-PCR) Analysis

Total RNA was extracted from dissected tissues of ten flies (equal number of males and females) and converted to cDNA using the FastGene Scriptase II cDNA Kit (LS53, Nippon Genetics Europe Co. Ltd., Düren, Germany). For real-time Q-PCR analysis, experiments were performed in triplicate using HOT FIREPol^®^ EvaGreen^®^ qPCR Mix Plus (08-36-00001, Solis BioDyne, Tartu, Estonia). Primers were designed using the primer-BLAST tool (http://www.ncbi.nlm.nih.gov/tools/primer-blast; accessed on 8 January 2018) and were as reported before [19]; RpL32/rp49 was used as a normalizer.

### 2.5. Preparation of Tissue Protein Extracts, Immunoblot Analysis, Measurement of ROS, and Proteasome Enzymatic Activities

Adult somatic (head and thorax of ten flies; equal number of males and females) or larvae (ten third-instar larvae) tissues were homogenized on ice in Nonident P-40 (NP-40) (74385, Sigma-Aldrich, St. Louis, MI, USA) lysis buffer (0.1% Nonidet P-40, 150 mM NaCl, 50 mm Tris/HCl buffer, pH 8.0) containing protease inhibitors (P1860, Sigma-Aldrich, St. Louis, MO, USA) and centrifuged for 10 min (4 °C) at 19,000× *g*. The protein content per sample was adjusted using a Bradford assay (Bio-Rad, Hercules CA, USA) and samples were analyzed with SDS-PAGE and immunoblotting. Twenty-five micrograms of protein was separated with sodium dodecyl sulfate-polyacrylamide gel electrophoresis (SDS-PAGE) and transferred to a nitrocellulose membrane (Bio-Rad, Hercules CA, USA). Following blocking with 5% nonfat dry milk, the membranes were incubated with the primary antibodies overnight at 4 °C, followed by the respective secondary antibodies. Immunoblots were developed using Clarity Western ECL Blotting Substrate (Bio-Rad, Hercules CA, USA). Blot quantification was performed through scanning densitometry, using ImageJ 1.53k (NIH). Protein expression levels were quantified vs. the respective controls set to 1.

ROS levels were measured as described previously [23]. Briefly, 10 flies (equal number of males and females) were incubated in CM-H2DCFDA (C6827, Thermo Fisher Scientific, MA, USA) dye for 30 min at 25 °C in the dark. Following centrifugation and dye removal, tissues were incubated for 10 min at 24 °C in PBS; samples were lysed in Nonidet P-40 lysis buffer (1% Nonidet P-40, 150 mM NaCl, and 50 mM Tris, pH 8.0) and cleared via centrifugation at 19,000× *g* for 10 min at 4 °C. The supernatant was diluted 1:4 (*v*/*v*) in ddH2O, and fluorescent dichlorodihydrofluorescein was recorded at excitation, 490 nm, and emission, 520 nm. Negative controls were unstained tissues incubated with only PBS buffer to detect autofluorescence.

For measuring proteasome peptidase activity, 10 dissected flies’ tissues (equal number of males and females) were lysed on ice in a buffer suitable for isolation of intact 26S proteasomes (0.2% NP-40, 5 mM ATP (fresh), 10% glycerol, 20 mM KCl, 1 mM EDTA, 1 mM DDT, and 20 mM Tris, pH 7.6). Protein content was adjusted with Bradford (Bio-Rad, Hercules CA, USA) and cleared supernatants were immediately used to determine the chymotrypsin-like (CT-L/LLVY) or caspase-like (C-L/LLE) proteasome proteolytic activities after recording (excitation, 350 nm; emission, 440 nm) the hydrolysis of the fluorogenic peptides Suc-Leu-Leu-Val-Tyr-AMC and Z-Leu-Leu-Glu-AMC, (Enzo Life Sciences, Farmingdale, NY, USA), respectively, at 37 °C for 30 min [24,25]. Equal numbers of male and female flies were used.

For all measurements, fluorescence was recorded in a Spark^®^ Tecan microplate reader (Tecan Group Ltd., Maennedorf, Switzerland).

### 2.6. Measurement of GLU/TREH Levels

GLU and TREH levels were measured as described previously [19], with minor modifications. Adult tissues of six flies, in triplicate, were homogenized in cold PBS for GLU or with trehalase buffer (5 mM Tris, pH 6.6, 137 mM NaCl, 2.7 mM KCl) for TREH measurement. The clear extract was then incubated for 10 min at 70 °C and a small amount was used for protein quantification via Bradford assay. Following centrifugation at max speed for 3 min, 30 μL of diluted (1/4) or undiluted for TREH measurement supernatant was transferred to a 96-well plate. The GLU assay was performed by adding in the sample 100 μL of GLU reagent (GAGO-20, Sigma-Aldrich, St. Louis, MI, USA) and incubating for 30 min at 37 °C. For TREH measurement, 100 μL of the GLU reagent (GAGO-20, Sigma-Aldrich, St. Louis, MI, USA) was added in the samples, which were then incubated for 18 h at 37 °C, followed by incubation (or not) with 0.05 U/mL of trehalase (T8778, Sigma-Aldrich, St. Louis, MI, USA). Absorbance was recorded at 540 nm and TREH levels were calculated after the subtraction of the GLU measurement at this step from the total amount of free GLU measured after TREH digestion. At least 3 replicates per genotype or experimental condition were performed.

### 2.7. Antibodies

Primary antibodies against the 20S-α (sc-65755), 26S p42A/Rpn7 (sc-65750), and 26S p54/Rpn10 (sc-65746) *Drosophila* proteasome subunits, along with the HRP-conjugated secondary antibodies, were from Santa Cruz Biotechnology, Inc. (Dallas, TX USA). The antibody against Gapdh (G9545) was from Sigma Aldrich (St. Louis, MI, USA) and the anti-Mouse-IgG Rhodamine (TRITC) conjugated antibody (715-025-151) was from Jackson ImmunoResearch (Europe LTD, Cambridge House, Ely CB7 4EX, UK). The p-Akt (pAkt1Ser505, phosphorylated at Ser505 Droso-Akt, active form; 4060), Akt (pan-Akt;4691), p-Gsk3 (pGsk3(α/β)Ser21/9, phosphorylated at Ser21/9 GSK-3a/b, inhibitory form; 9331S), and β-Actin (4967S) antibodies were from Cell Signaling Technology, Inc. (Danvers, MA, USA). The Gsk3 (anti-GSK3, clone 4G-1E; 05-412) antibody was from Millipore (Merck KGaA, Darmstadt, Germany) and the anti-Atp5a antibody (complex V subunit-ATP5A; ab14748) was purchased from Abcam (Cambridge, UK); the antibody against PSMD11/Rpn6 (NBP1-46191) was from Novus Biologicals. The anti-GLY antibody was a gift from Prof. Otto Babba (Ohu University, Japan). Bodipy™ 493/503 dye (D3922) and DAPI (D1306) were purchased from Molecular Probes™ (Thermo Fisher Scientific Inc., Waltham, MA, USA). 

### 2.8. CSLM and Immunofluorescence Staining

Dissections, fixation, and immunostaining of tissues were performed as described before [25]. In brief, third-instar larvae and/or adults were dissected in PBS. Tissues were fixed in 4% formaldehyde for 15 min, washed in PBS containing 0.1% Triton X-100, and then incubated with the primary antibody overnight at 4 °C. Secondary antibodies were applied for 1 h at RT. After 3 washes with PBS, samples were mounted in Mowiol^®^ 4–88 (4–88, Sigma-Aldrich, St. Louis, MO, USA) and viewed in a Digital Eclipse Nikon C1 (Nikon, Melville, NY, USA) CLSM equipped with 40× 1.0 NA differential interference contrast (DIC) and 60× 1.4 NA DIC Plan Apochromat objectives; image capturing was performed using the EZC1 acquisition and images were analyzed with the CLSM software version EZ-C1 (Nikon Inc., Tokyo, Japan).

### 2.9. Statistical Analyses

The presented experiments were analyzed at least in triplicate, unless otherwise indicated. Data points correspond to the means of the independent experiments; error bars denote standard deviation (SD), and differences between compared groups were evaluated using the independent (unpaired) *t*-test analysis or a two-way ANOVA test followed by Tukey’s multiple-comparisons test. For graphical representation of data and statistical analyses, MS Excel (Microsoft Office, Washington, DC, USA), the Statistical Package for Social Sciences (IBM SPSS, version 23.0 for Windows, New York, NY, USA), and GraphPad Software (Prism 8.00, GraphPad Software Inc., Boston, MA, USA) were used. Significance was accepted at * *p* < 0.05 (shown in graphs by one asterisk); two asterisks denote ** *p* < 0.01. Gene expression was plotted vs. the respective control set to 1.

## 3. Results

### 3.1. ImpL2 Knockdown (KD) Activates IIS-, Antioxidant- and Proteostasis-Related Modules

**Figure 1:**
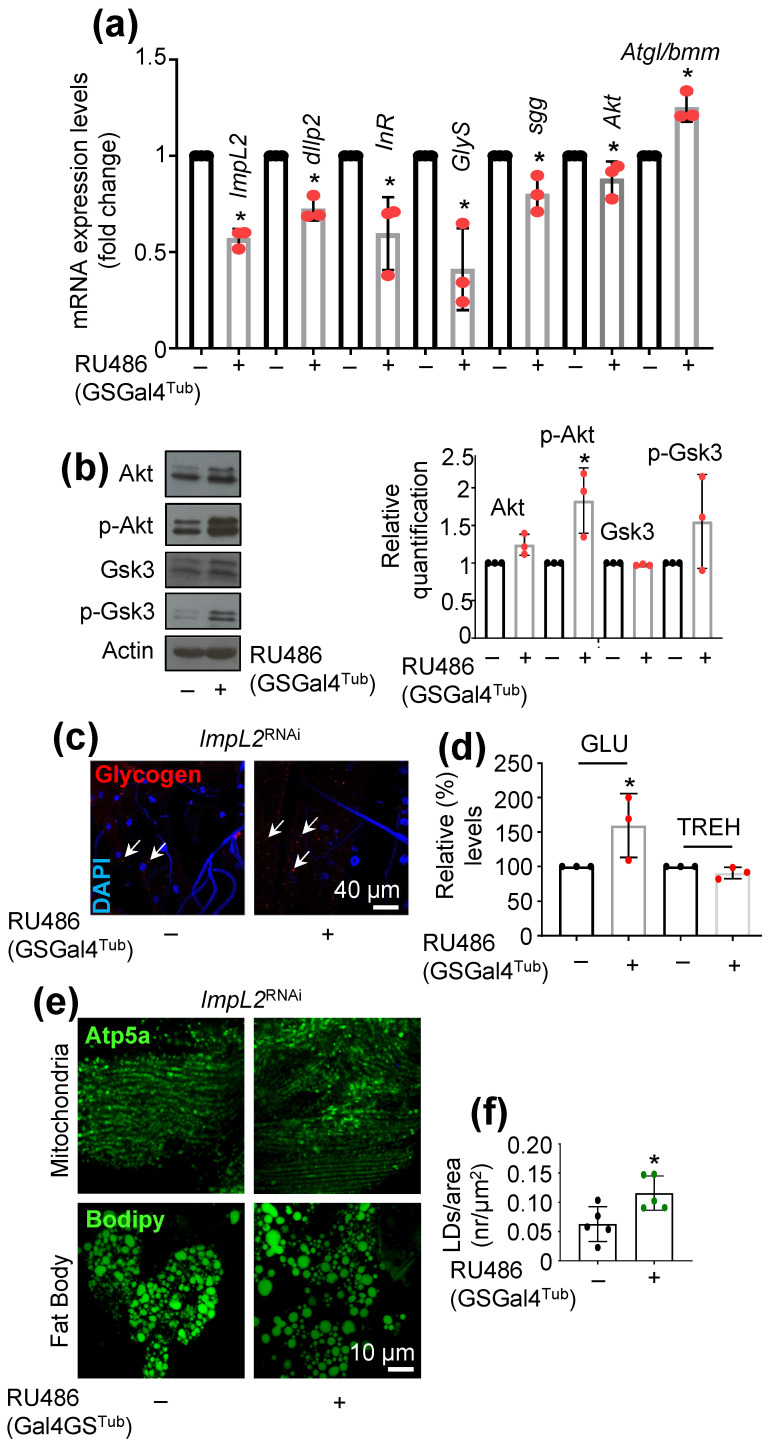
*ImpL2* downregulation in *Drosophila* activates IIS. (**a**) Relative mRNA expression of *ImpL2*, *dIlp2*, *InR*, *sgg*, *Akt*, *GlyS*, and *Atgl/bmm* genes after ubiquitous downregulation of *ImpL2*. (**b**) Immunoblot analyses and relative immunoblotting quantification (*n* = 3) of tissue protein samples probed with antibodies against Akt, p-Akt, Gsk3, and p-Gsk3 after ubiquitous KD of *ImpL2*. (**c**) Confocal imaging of GLY (red, indicated by the arrows) immunofluorescence staining in larvae muscle tissues. Nuclei were stained with DAPI (blue). (**d**) Relative content of GLU and TREH in flies’ somatic tissues after *ImpL2* KD (**e**) Confocal imaging of mitochondria (Atp5a) and fat body (Bodipy staining) of adult flies after *ImpL2* KD. (**f**) Lipid droplets quantification (number/area) of the shown genotypes. Flies were exposed to 320 μM RU486 for 7 days. Gene expression (**a**) was plotted vs. control set to 1 (*RpL32/rp49* gene was used as reference). Actin (**b**) probing was used to demonstrate equal protein loading. Bars, ±SD; *n* = 3, * *p* < 0.05.

To understand the impact of ImpL2 on IIS regulation in the fly model, we ubiquitously KD *ImpL2* (*ImpL2*^RNAi^) by using the GSGal4Tub driver. Initially, we assayed the expression levels of major IIS modules in induced (flies fed with RU486) or control (non-induced flies) *Drosophila* adult flies. Apart from *ImpL2* downregulation, we found reduced expression levels in flies’ somatic tissues for the majority of analyzed genes (i.e., *dIlp2*, *InR*, *shaggy/glycogen synthase kinase* 3 (*sgg/Gsk3*), *glycogen synthase* (*GlyS*), and *serine/threonine kinase* (*Akt*)) except from *Atgl/bmm* (*brummer*), which is involved in lipogenesis and was found to be upregulated after *ImpL2* KD (Figure 1a). Further, protein expression analyses showed that *ImpL2* KD resulted in increased Akt and p-Akt (phosphorylated Akt, active form) expression levels along with increased expression of p-Gsk3 (phosphorylated Gsk3, inactive form) (Figure 1b). The imaging of transgenic *ImpL2* KD third-instar larvae revealed the accumulation of GLY in muscle fibers (Figure 1c); this effect was accompanied with increased GLU, but not trehalose (TREH; a hemolymph circulating sugar), in adult flies’ somatic tissues (Figure 1d). To further examine the metabolic state of tissues, we analyzed muscle fibers’ mitochondrial and fat body’s lipids content after *ImpL2* KD in flies. We observed no differences in muscle fiber mitochondria (Figure 1e), whereas we found a significant increase in lipid droplet number in adult flies’ fat bodies (Figure 1e,f), a tissue known to affect insulin-producing cells’ (IPCs) activity [26]. Overall, our data suggest that *ImpL2* KD in adults flies activates IIS and anabolic pathways.

**Figure 2:**
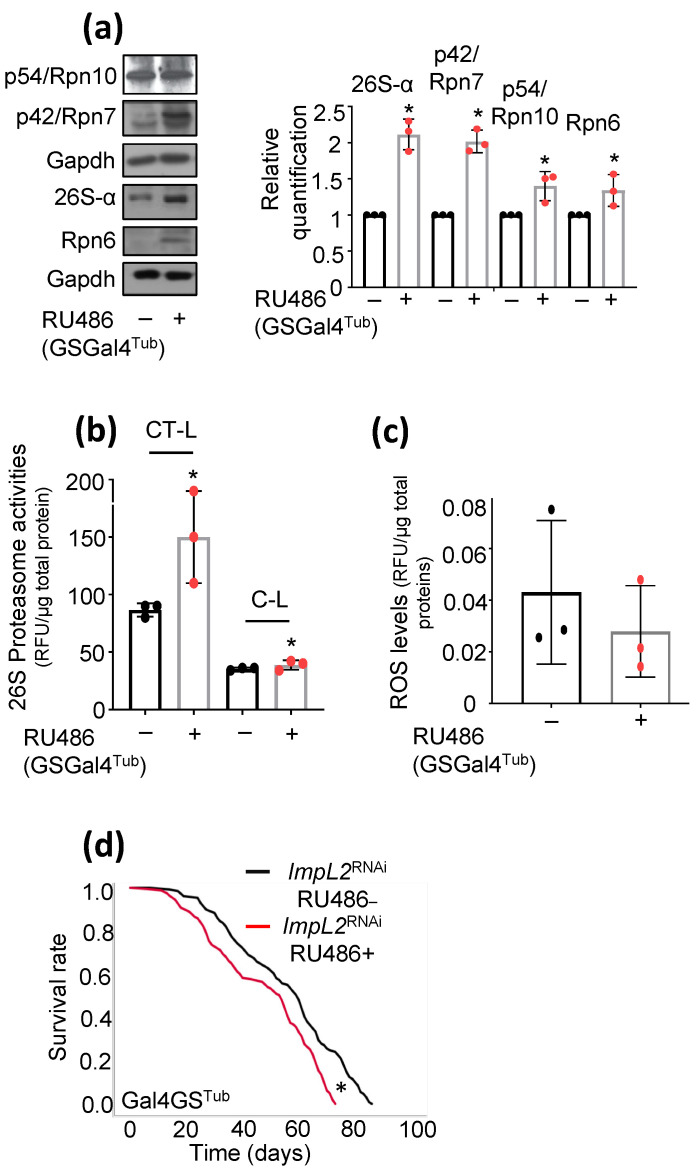
KD of *ImpL2* increases proteasome subunits expression and peptidases activity; it also mildly decreases flies’ longevity. (**a**) Immunoblot analyses and relative immunoblotting quantification (*n =* 3) of tissue protein samples probed with antibodies against proteasomal subunits p54/Rpn10, p42/Rpn7, 26S-α, and Rpn6 in flies’ tissues after *ImpL2* KD. (**b**) Chymotrypsin (CT-L)- and caspase (C-L)-like activities of 26S proteasome after *ImpL2* KD. (**c**) ROS levels in flies’ somatic tissues after *ImpL2* KD. (**d**) Longevity curves of *ImpL2* KD flies vs. control flies (log-rank, Mantel–Cox tests: non-induced flies vs. *ImpL2*^RNAi^-induced flies *p* < 0.0001). In (**a**–**c**), flies were exposed to 320 μM RU486 for 7 days. Gapdh probing (**a**) was used to demonstrate equal protein loading. Bars, ±SD; *n* = 3, * *p* < 0.05.

We next asked whether *ImpL2* KD affects proteostatic and/or antioxidant modules. We found that partial loss of *ImpL2* functionality led to increased expression of proteasome protein subunits (Figure 2a) and of chymotrypsin-like (CT-L) and caspase-like (C-L), peptidase activities (Figure 2b); also, we noted decreased reactive oxygen species (ROS) levels after *ImpL2* KD in flies’ tissues (Figure 2c), indicating the activation of antioxidant responses, parallel to the ubiquitin proteasome pathway (UPP). Since the mild upregulation of stress responses and UPP has been associated with the amelioration of age-related phenotypes [27], and IIS is one of the most highly conserved signaling pathways that modulates longevity across species [28], we next assessed the effect of *ImpL2* KD on flies’ longevity and found, in this experimental setting, a marginal decrease in flies’ longevity (Figure 2d).

Collectively, these findings suggest that inhibiting ImpL2 activity triggers the activation of both IIS and antioxidant/proteostatic pathways; yet prolonged *ImpL2* KD mildly decreases flies’ lifespan. This finding highlights the significance of IIS fine-tuning for sustaining homeostasis and facilitating a state of healthy aging.

### 3.2. Proteostatic Responses Remain Upregulated after Concomitant ImpL2 KD in cncC^OE^ Flies

**Figure 3:**
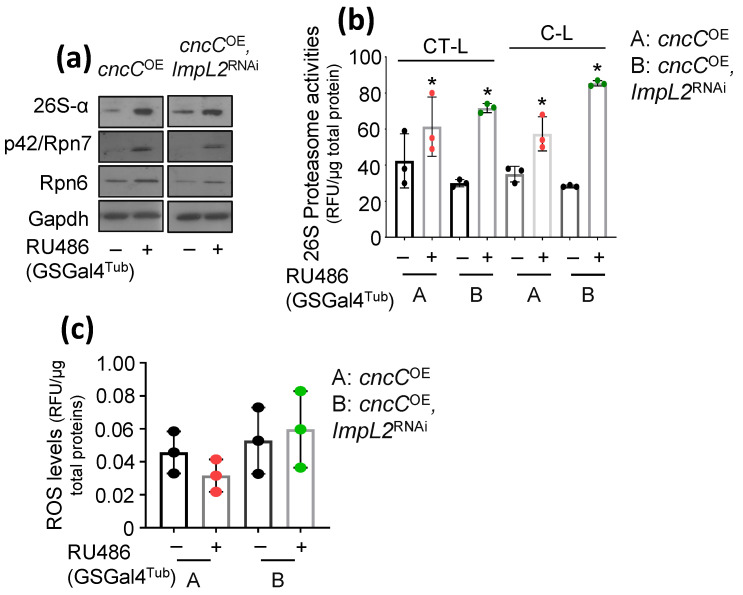
Concomitant *ImpL2* KD in *cncC^OE^* transgenic flies does not affect *cncC^OE^*-mediated UPP activation. (**a**) Representative (*n* = 3) immunoblot analyses of tissue protein samples from the shown genotypes probed with antibodies against proteasomal subunits 26S-α, p42/Rpn7, and Rpn6. (**b**) Chymotrypsin (CT-L)- and caspase (C-L)-like 26S proteasome activities. (**c**) ROS levels of the shown genotypes. In (**a**–**c**), flies were exposed to 320 μM RU486 for 7 days. Gapdh probing (**a**) was used to demonstrate equal protein loading. Bars, ±SD; *n* = 3, * *p* < 0.05.

We recently found that sustained ubiquitous *cncC^OE^* in adult flies accelerates aging due to metabolic deregulation being evident by hyperglycemia and extensive lipolysis. We hypothesized that these phenotypes may also relate to increased ImpL2 activity; an IIS inhibitor and a cncC/Nrf2 transcriptional target [19]. We thus KD *ImpL2* in *cncC^OE^* flies, using the GSGal4Tub driver, and found that this genetic intervention increased both proteasomal protein subunits expression levels (Figure 3a) and proteasomal peptidase activities, vs. solely *cncC^OE^* flies (Figure 3b), suggesting that UPP remains highly active likely as a result of enhanced IIS signaling (see above; [19]). Notably, the measurement of transgenic flies’ oxidative load showed that *ImpL2* KD in *cncC^OE^* flies nulled the cncC/Nrf2 impact on the cellular oxidative load (Figure 3c). Thus, *ImpL2* KD in the *cncC^OE^* background maintains increased protein synthesis quality control mechanisms, i.e., the ubiquitin proteasome pathway, in flies’ tissues.

### 3.3. Concomitant to cncC^OE^, ImpL2 KD Partially Restores IIS Activity and Attenuates the Sustained cncC^OE^-Induced Metabolic Deregulation, Leading to Lifespan Extension

**Figure 4:**
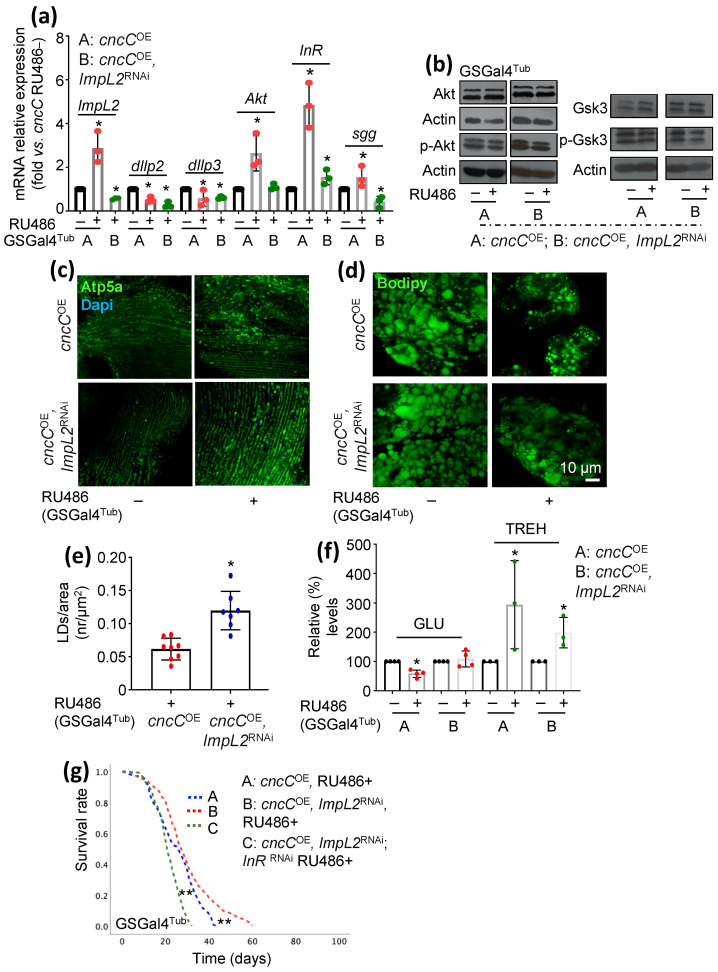
*ImpL2* KD partially reverts sustained *cncC^OE^*-mediated toxic phenotypes. (**a**) Relative mRNA expression of *ImpL2*, *dIlp2*, *dIlp3*, *Akt*, *InR*, and *sgg*/*Gsk3* genes after ubiquitous KD of *ImpL2* in *cncC^O^*^E^ flies. (**b**) Immunoblot analyses of tissue protein samples probed with antibodies against p-Akt, Akt, Gsk3, and p-Gsk3 after ubiquitous *ImpL2* KD in *cncC^OE^* flies. (**c**) Confocal imaging of muscle mitochondria (Atp5a) and (**d**) of adult flies’ fat body lipid droplets (Bodipy). Nuclei were stained with DAPI (blue). (**e**) Lipid droplet quantification (number/area) of the indicated genotypes. (**f**) Relative content of GLU and TREH in somatic tissues of *cncC^OE^* or *cncC^O^*^E^, *ImpL2*^RNAi^ adult flies. (**g**) Longevity curves of flies with the shown genotypes (log-rank, Mantel–Cox tests: *cncC^OE^* RU486+ vs. *cncC^OE^*, *ImpL2*^RNAi^ RU486+ *p* < 0.000; *cncC^OE^* RU486+ vs. *cncC^OE^*, *ImpL2*^RNAi^, *InR*^RNAi^ RU486+ *p* < 0.000; *cncC^OE^*, *ImpL2*^RNAi^ RU486+ vs. *cncC^O^*^E^, *ImpL2*^RNAi^; *InR*^RNAi^ RU486+ *p* < 0.000). Gene expression (**a**) was plotted vs. control set to 1 (*RpL32/rp49* gene was used as reference). Actin probing (**b**) was used to demonstrate equal protein loading. Flies in (**a**–**f**) were exposed to 320 μM RU486 for 7 days. Bars, ±SD; *n* = 3, * *p* < 0.05; ** *p* < 0.01.

To assay the regulatory action of *ImpL2* KD on sustained *cncC^OE^*-induced metabolic/diabetic phenotypes, we analyzed the expression levels of major IIS components in double-transgenic flies, i.e. *cncC^OE^* and *ImpL2*^RNAi^ vs. sole *cncC^OE^* flies. We found that while sole *cncC^OE^* increased the expression of most (except of *dIlp2* and *dIlp3*) of the IIS-related genes assayed, concomitant *ImpL2*^RNAi^ reverted this pattern (Figure 4a). In addition, *ImpL2* KD increased the expression levels of p-Akt and of (inhibitory) p-Gsk3 vs. *cncC^OE^* flies, suggesting IIS reactivation (Figure 4b and Figure S1a).

We then asked whether *ImpL2* KD could rescue the detrimental metabolic phenotypes manifested in *cncC^OE^* flies [19]. Mitochondria staining in flies’ tissues revealed that *ImpL2* KD in *cncC^OE^* flies triggered a denser mitochondrial network (Figure 4c) and reduced *cncC^OE^*-mediated lipolysis as it increased lipid droplet content in the fat body (Figure 4d,e). Moreover, by analyzing sugar levels in flies’ tissues, we found that *ImpL2* KD tended to restore more physiological GLU levels; this findings coincides with reduced TREH (composed from 2 glucose molecules) levels in flies’ tissues (Figure 4f). Consistently, double-transgenic flies not only had a better health span but also lived longer than *cncC^OE^* flies (Figure 4g).

Given that *ImpL2* KD partially rescued both metabolic deregulation and accelerated aging in *cncC^OE^* flies, we then examined whether these beneficial effects were mediated through IIS reprogramming. Thus, we ubiquitously downregulated both *ImpL2* (*ImpL2*^RNAi^) and *InR* (*InR*^RNAi^) in *cncC^OE^* flies and found that *InR* KD in the *ImpL2*^RNAi^, cncC^OE^ background abolished the *ImpL2*^RNAi^ mediated beneficial effects (Figure 4g and Figure S1b). Thus, the rescue effects of *ImpL2* KD in *cncC^OE^* flies are likely mediated by partial IIS reactivation.

Altogether, these results demonstrate that the ImpL2-mediated reduction in IIS signaling in *cncC^OE^* flies, as part of build-in negative feedback loops aiming to suppress cncC/Nrf2 activity [19], plays an important role in the manifestation of several diabetes-like metabolic alterations after prolonged cncC/Nrf2 overactivation.

### 3.4. Treatment of cncC^OE^ Flies with the Anti-Diabetic Drug Metformin (Met) Partially Restores the Functionality of Metabolic/Mitostatic Pathways and Extends Flies’ Lifespan

**Figure 5:**
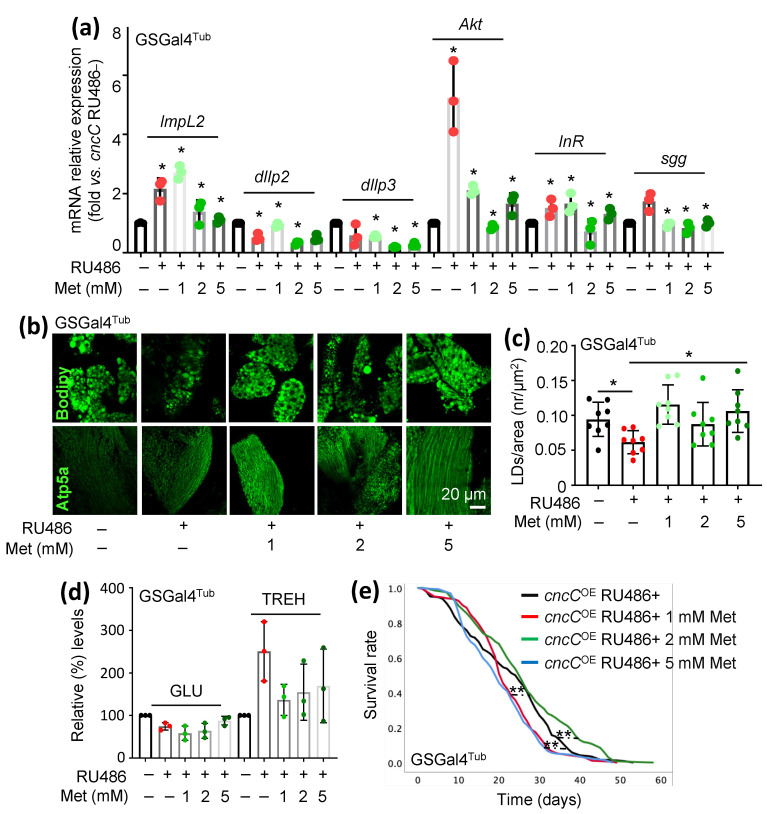
Met treatment partially suppresses the metabolic phenotypes induced in flies by sustained *cncC^OE^*. (**a**) Relative mRNA expression of *ImpL2*, *dIlp2*, *dIlp3*, *Akt*, *InR*, and *sgg/Gsk3* genes after treatment with Met (metformin) at the indicated concentrations. (**b**) Confocal imaging of fat body (Bodipy) and muscle mitochondria (Atp5a) of adult flies of the described genotypes after Met treatment. (**c**) Lipid droplet quantification (number/area) of the shown genotypes. (**d**) Relative content of GLU and TREH levels (vs. non-treated *cncC^OE^*) in somatic tissues of *cncC^OE^* flies after Met treatment. (**e**) Longevity curves of *cncC^OE^* adult flies after treatment with Met at the indicated concentrations (log-rank, Mantel–Cox tests: *cncC^OE^* vs. *cncC^OE^* 1 mM Met *p* < 0.007; *cncC^OE^* vs. *cncC^OE^* 2 mM Met *p* < 0.001; *cncC^OE^* vs. *cncC^OE^* 5 mM Met *p* < 0.000). In (**a**–**d**), flies were exposed to 320 μM RU486 for 7 days. In (**a**–**d**), Met treatment was applied for 7 days, starting from day 1 of *cncC* transgene induction. Gene expression (**a**) was plotted vs. control set to 1 (*RpL32/rp49* gene was used as reference). Bars, ±SD; *n* ≥ 3, * *p* < 0.05; ** *p* < 0.01.

Met is a biguanide drug and one of the most widely used oral medications for reducing blood GLU levels [29]. Studies on a variety of model organisms have shown that Met treatment also has beneficial effects on health and survival rates [30]. Given these beneficial effects of Met in metabolic reprogramming, we then treated *cncC^OE^* flies with a broad range of Met concentrations. We found that, in this experimental context, Met affected IIS-related genes in a dose-dependent manner and that 2 mM or 5 mM of Met reduced the expression levels of the *cncC*, *ImpL2*, *dIlp2*, and *dIlp3* genes vs. *cncC^O^*^E^ non-Met-treated flies (Figure 5a). Notably, similarly to what was found after *ImpL2* KD, Met also reduced the expression levels of *Akt*, *InR*, and *sgg* genes (Figure 5a). Exposure of *cncC^OE^* flies to Met also improved the thoracic muscles’ mitochondrial network (Figure 5b) and restored fat bodies’ lipid content at all concentrations used (Figure 5b,c). In addition, Met treatment largely normalized GLU and TREH levels in *cncC^OE^* flies’ tissues (Figure 5d).

Considering that metabolic deregulation starts to be evident in transgenic flies at about 1 week post *cncC* overexpression [19], we started flies’ treatment with Met at day 7 post cncC transgene induction. Specifically, adult flies (7 days old) in this experimental “therapeutic” protocol were treated with 1, 2, or 5 mM of Met for 3 days. Our data showed that Met reduced lipolysis in the fat body (Figure S2a) and normalized GLU levels in the *cncC^OE^* flies’ tissues dose-dependently (Figure S2b). Finally, we found that treatment with 1 or 2 mM Met improved *cncC^OE^* flies’ health span (Figure 5e); similarly, starting Met administration at day 7 post cncC induction improved flies’ health span in all concentrations used, suggesting the potential advantageous outcomes of Met administration parallel to phenotype appearance (“symptoms” initiation) rather than during early adulthood (Figure S2c).

Taken together, these data suggest that Met treatment can partially rescue metabolic deregulation caused by *cncC^OE^*-mediated sustained organismal stress. This finding further supports the possible therapeutic role of Met in attenuating stress-induced metabolic and/or diabetic phenotypes.

## 4. Discussion

We reported recently that the overexpression of *cncC* in *Drosophila melanogaster* tissues results in ImpL2 accumulation in adult flies’ brain and hemolymph, while ChIP analyses revealed the binding of cncC on ImpL2-regulatory elements, indicating a direct positive regulation of ImpL2 by cncC [19]. Thus, ImpL2 upregulation and consequent IIS suppression in *cncC^OE^* flies is likely involved in the metabolic disruption and tissue deterioration seen after sustained *cncC* overexpression; *ImpL2* upregulation in *cncC^OE^* flies is part of a negative feedback loop aiming to suppress cncC activity [19]. Our shown data here indicate that the downregulation of *ImpL2* in *Drosophila* flies results in IIS activation.

PI3K/Akt activation has been found to be essential for Nrf2 nuclear translocation [31]. In line with this, *ImpL2* KD in flies reduced ROS levels. Moreover, *ImpL2* KD induced high rates of proteasome activity and high expression of proteasomal proteins, suggesting UPP upregulation likely via Nrf2 activation; in support, *ImpL2* KD in *cncC^OE^* flies resulted in sustained high levels of UPP activity. Beyond the activation of a wide range of cytoprotective targets, Nrf2 also plays a crucial role in regulating mitochondrial biogenesis; in maintaining mitochondrial structural integrity and in ensuring proper mitochondrial function [32]. Reportedly, *Drosophila* cncC/Nrf2 regulates mitochondrial dynamics, energetics, O_2_ consumption, ATP production, assembly of OXPHOS machineries, and mitochondrial genes expression [19]. Beyond the direct action of cncC/Nrf2 in mitochondria, *ImpL2* KD in *cncC^OE^* flies improved mitochondrial networks, likely also via IIS reactivation.

The upregulation of cncC alters flies’ metabolic state, leading to high levels of circulating sugars, extensive lipolysis, and diabetes-like phenotypes [19]. Notably, the downregulation of *ImpL2* in *cncC^OE^* flies reduced TREH levels in flies’ tissues, improved the mitochondrial network, and suppressed lipolysis in the fat body. The *Drosophila* fat body stores energy in the form of triglycerides, while it is involved in the regulation of metabolism, the storage of overabundant GLU in the form of GLY, and in sensing nutrients availability. Moreover, similarly to mammals, IIS in *Drosophila* promotes fat storage in the adipose tissue [33]. Recently, studies in *Drosophila* cancer models showed that ImpL2 mediates tissue-specific cancer cachexia [34,35,36], a phenotype similar to that seen after sustained *cncC^OE^* [19]. Remarkably, cancer-related metabolic traits and diabetic phenotypes share many similarities; cachexia, which is a cancer-related wasting syndrome, is also characterized by metabolic aspects that include alternations in GLU, lipid, protein, and energy metabolism [37]. Overall, our data further support a role for ImpL2 in reducing systemic IIS; affecting flies’ metabolic state and consequently in modulating their health span/lifespan.

Interestingly, Met treatment partially rescued *cncC^OE^*-mediated metabolic deregulation. Met is widely used for the treatment of diabetes and other age-related diseases [38,39]. However, the mechanistic details of Met action remain only partially understood and are sometimes even controversial. Reportedly, Met inhibits mitochondria complex 1, resulting in ATP depletion, cellular AMP elevation, and the activation of AMPK (5’ AMP-activated protein kinase), a master cellular energy sensor [40,41]. However, Met may also act in an AMPK-independent manner [42,43,44].

Similarly to previous studies [45], our data indicate that Met affected IIS modules (such as Akt protein expression) dose-dependently. Reportedly, high concentrations of Met are needed in isolated mitochondria for the direct inhibition of mitochondrial complex I, which is thought be a primary target of Met, while micromolar concentrations are sufficient to achieve dose- and time-dependent mild, reversible, and selective complex I inhibition [46]. Consistently, we found Met to alter *Drosophila* GLU levels in *cncC^OE^* flies dose-dependently. It is important to note that Met does not cause hypoglycemia *per se* in mammals but it rather reduces hepatic GLU production through improved hepatic insulin sensitivity, which then results in a reduction in fasting plasma GLU levels [47]. Remarkably, we found TREH levels to be decreased and the somatic GLU levels to be increased after the treatment of *cncC^OE^* flies with Met; TREH is main circulating disaccharide in flies, synthesized through the condensation of two glucose molecules, a process catalyzed by the enzyme trehalose-6-phosphate synthase (Tps1) [48]. In addition, Met treatment tended to increase the mitochondria number in the thoracic flight muscle and suppressed lipolysis in the fat body; additionally, Met is thought to lower the mitochondria oxidative load and induce mitochondrial biogenesis via PGC-1a [49,50]. Finally, Met treatment increased (at specific doses) *cncC^OE^* flies’ health span and extended their lifespan. While studies on model organisms have revealed that the lifespan-extending and/or health span-improving properties of Met rely on its ability to suppress major hallmarks of biological aging [51], several experimental and clinical observations establish its ability as a gero-therapeutic agent [47]. Accordingly, our data indicate that the “therapeutic” treatment of *cncC^OE^* flies with Met in a later life stage, in which *cncC^O^*^E^-mediated phenotypes are exaggerated, is critical for the promotion of healthy aging.

Taken together, our study provides further insights into the mechanism through which persistent cncC overactivation induces metabolic deregulation in the circulation and somatic tissues of flies. Moreover, these observations exemplify the effect of chronic stress in predisposition to diabetic phenotypes, indicating the potential prophylactic role of maintaining (e.g., via Metformin treatment) normal IIS functionality.

## 5. Conclusions

*cncC^OE^* in *Drosophila* among others suppresses insulin signaling and consequently triggers diabetes-like phenotypes. Here, we investigated the association between ImpL2 activity and *cncC^OE^*-induced metabolic changes. Our observations revealed that decreasing ImpL2 levels mitigated stress-induced diabetic phenotypes and extended the lifespan of *cncC^OE^* flies, suggesting that ImpL2 plays a significant role in initiating the metabolic deregulation associated with prolonged *cncC^OE^*. In addition, we found that treatment with the antidiabetic drug metformin could also improve *cncC^OE^* metabolic phenotypes and increase *cncC^OE^* flies’ longevity. Our research provides valuable insights into the mechanisms through which sustained *cncC^OE^* contributes to tissue and metabolic alterations.

## Data Availability

The datasets generated during and/or analyzed during the current study are available from the corresponding author on reasonable request.

## Supplementary Materials

The following supporting information can be downloaded at: https://www.mdpi.com/article/10.3390/cells12222650/s1, Figure S1: Relative western blots quantification and longevity assays; Figure S2: Metabolic alterations after Met treatment of *cncC^OE^* transgenic flies.
