# Supplementary material for: Sustained Nrf2 Overexpression-Induced Metabolic Deregulation Can Be Attenuated by Modulating Insulin/Insulin-like Growth Factor Signaling
Source: Cells. 2023 Nov 18;12(22):2650. doi: 10.3390/cells12222650 (PMC10670064; doi:10.3390/cells12222650)
Supplement: Supplementary file 1 [file cells-12-02650-s001.zip › cells-2683223-supplementary.pdf]

Sustained Nrf2 overexpression-induced metabolic deregulation can be attenuated by modulating the insulin/insulin-like growth factors signaling

Sentiljana Gumeni, Maria Lamprou, Zoi Evangelakou, Maria S. Manola, Ioannis P. Trougakos

Supporting Material

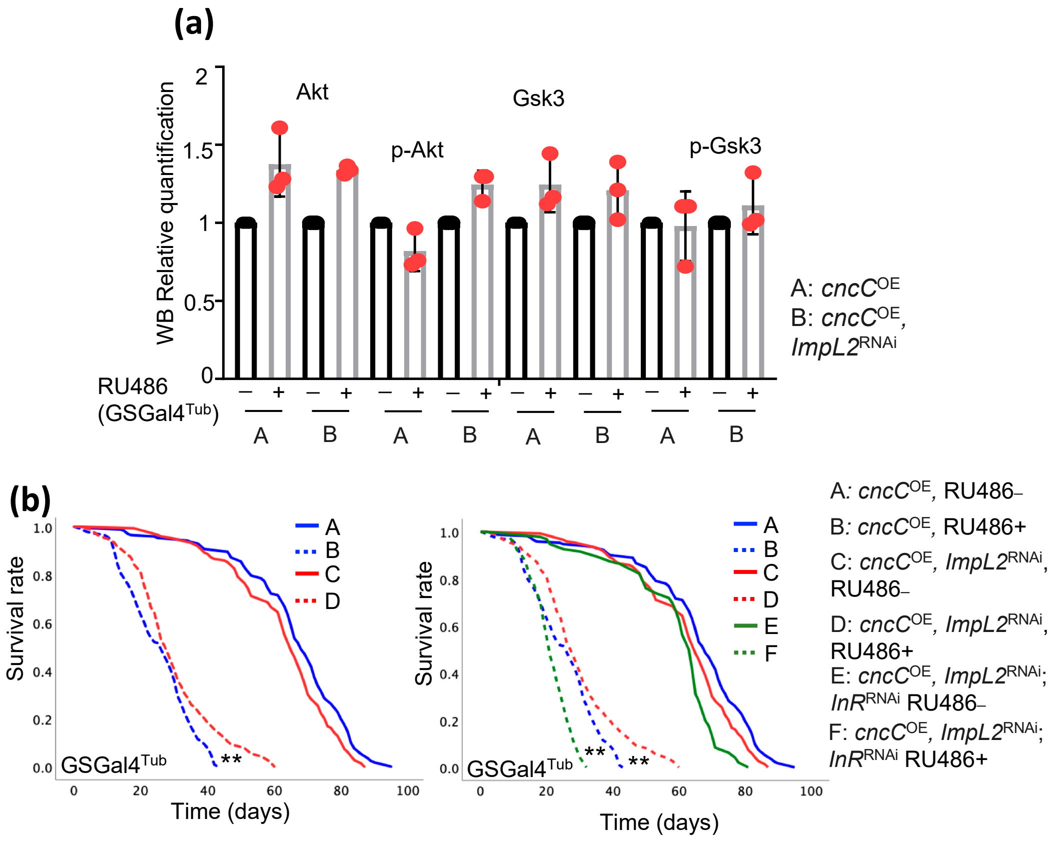

Figure S1. Relative western blots quantification and longevity assays. (a) Relative immunoblotting quantification (*n*=3) of tissue protein samples probed with antibodies against p-Akt, Akt, Gsk3, p-Gsk3, after ubiquitous downregulation (or not) of *ImpL2* in *cncC*^{OE} flies. (b) Longevity curves of the described genotypes. Log-rank, Mantel-Cox test: *cncC*^{OE} RU486- *vs* *cncC*^{OE} RU486+ P < 0.000; *cncC*^{OE}, *ImpL2*^{RNAi} RU486- *vs* *cncC*^{OE}, *ImpL2*^{RNAi} RU486+ P < 0.000; *cncC*^{OE} RU486+ *vs* *cncC*^{OE}, *ImpL2*^{RNAi} RU486+ P < 0.000; *cncC*^{OE}, *ImpL2*^{RNAi}, *InR*^{RNAi} RU486- *vs* *cncC*^{OE}, *ImpL2*^{RNAi}, *InR*^{RNAi} RU486+ P < 0.000; *cncC*^{OE} RU486+ *vs* *cncC*^{OE}, *ImpL2*^{RNAi}, *InR*^{RNAi} RU486+ P < 0.000; *cncC*^{OE}, *ImpL2*^{RNAi} RU486+ *vs* *cncC*^{OE}, *ImpL2*^{RNAi}; *InR*^{RNAi} RU486+ P < 0.000.

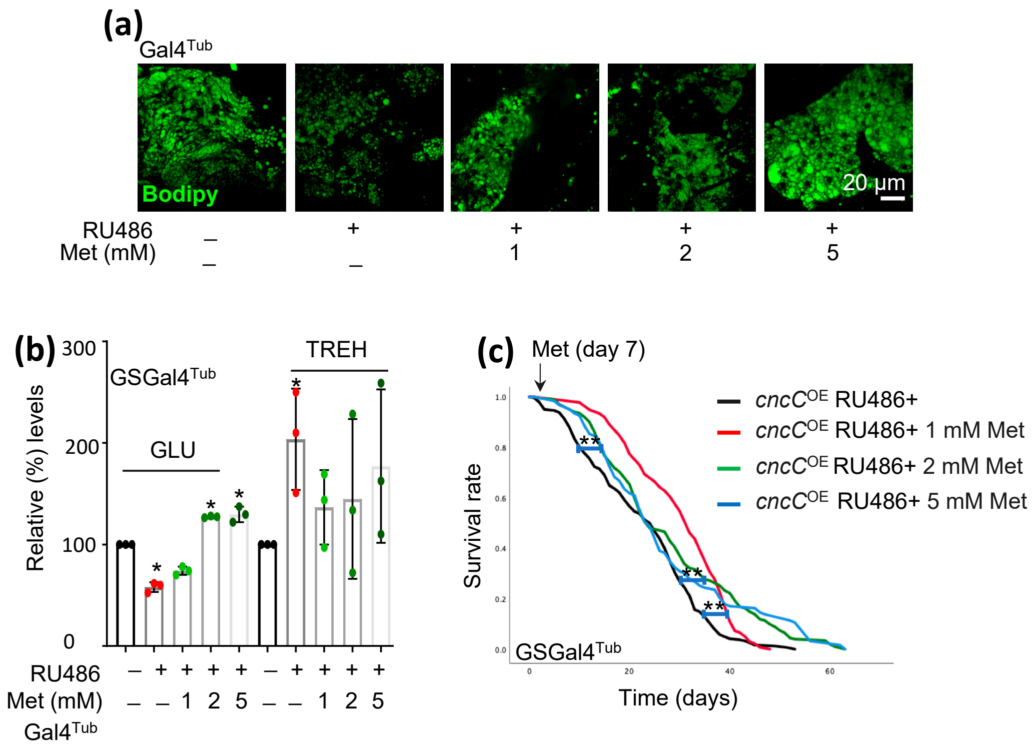

Figure S2. Metabolic alterations after Met treatment of *cncC*^{OE} transgenic flies. (a) CLSM visualization of *cncC*^{OE} fat body (Bodipy/green) lipid droplets after treatment (or not) with Met at different concentrations. (b) GLU and TREH levels (% *vs* non-treated *cncC*^{OE} flies) in somatic tissues of *cncC*^{OE} flies following Met treatment. (c) Longevity curves of *cncC*^{OE} adult flies treated with the indicated concentrations of Met for 7 days (starting point of treatment at day 7 is indicated in the graph with the arrow) post-induction of *cncC*^{OE} (Log-rank, Mantel-Cox test: *cncC*^{OE} *vs cncC*^{OE} 1mM Met P <0.000; *cncC*^{OE} *vs cncC*^{OE} 2mM Met P <0.000; *cncC*^{OE} *vs cncC*^{OE} 5mM Met P <0.000). In (a, b) flies were exposed to 320 μM RU486 for 10 days; Met treatment was applied for 3 days starting from day 7 post-*cncC*^{OE} induction. Bars, ±SD; n=3; **P < 0.01.

Supplementary list of genes assayed

Akt (akt kinase, FBgn0010379, CG4006); *bmm* (brummer/Atgl, FBgn0036449, CG5295); *cncC* (cap-n-collar isoform-C, FBgn0262975, CG43286); *Ilp2* (Insulin-like peptide 2, FBgn0036046, CG8167); *Ilp3* (Insulin-like peptide 3, FBgn0044050, CG14167); *ImpL2* (Ecdysone-inducible gene L2, FBgn0001257); *InR* (Insulin-like receptor, FBgn0283499, CG18402); *Keap1* (Kelch-like ECH-associated protein 1FBgn0038475, CG3962); *Prosα7* (Proteasome α7 subunit, FBgn0023175, CG1519); *Prosβ5* (Proteasome β5 subunit, FBgn0029134, CG12323); *RpL32/rp49* (Ribosomal protein L32, FBgn0002626, CG7939); *Rpn6* (Regulatory particle non-ATPase 6, FBgn0028689, CG10149); *Rpn11* (Regulatory particle non-ATPase 11, FBgn0028694, CG18174); *sgg* (shaggy/GSK3, FBgn0003371, CG2621); *GlyS* (Glycogen synthase, FBgn0266064, CG6904).
